# Supplementary material for: Overestimation of the effect of climatic warming on spring phenology due to misrepresentation of chilling
Source: Nat Commun. 2020 Oct 2;11:4945. doi: 10.1038/s41467-020-18743-8 (PMC7532433; doi:10.1038/s41467-020-18743-8)
Supplement: Supplementary file 1 — Supplementary Information [file 41467_2020_18743_MOESM1_ESM.pdf]

Supplementary Information

Overestimation of the effect of climatic warming on spring phenology due to misrepresentation of chilling

Wang et al.

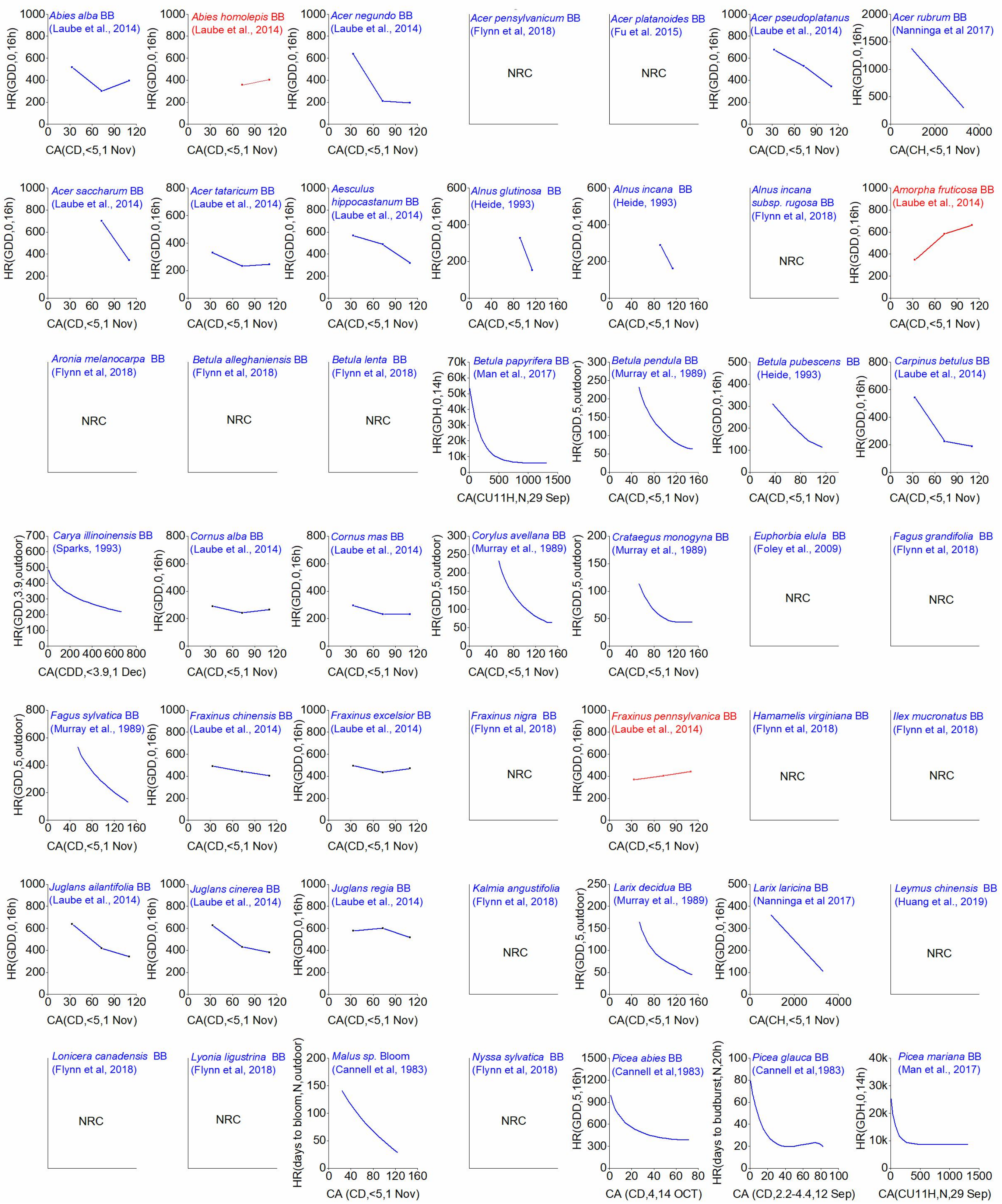

Supplementary Fig. 1 partial

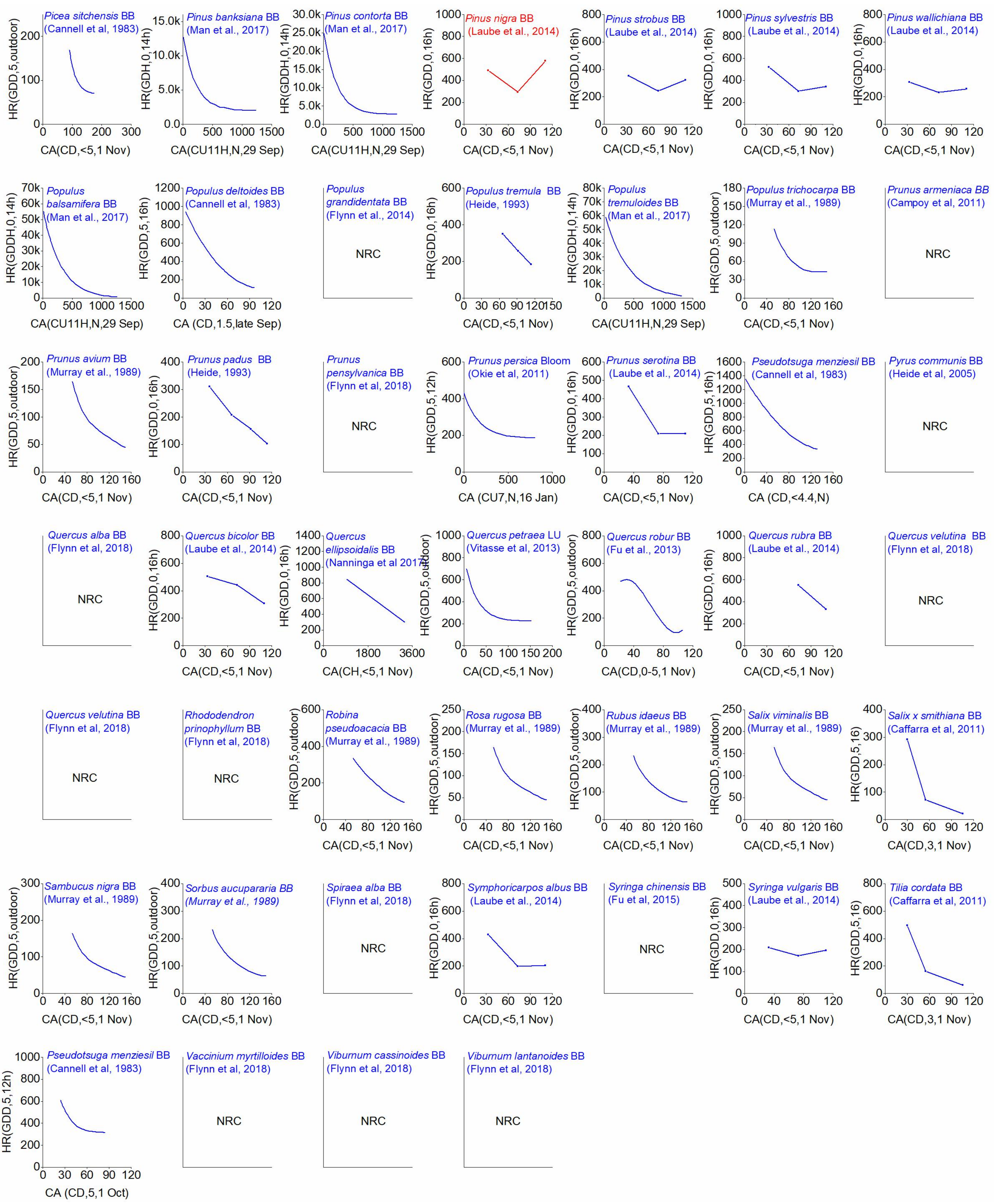

Supplementary Fig. 1 | Relationship between heat requirement (HR) of spring phenology and chilling accumulation (CA) for 95 perennials. The references (Supplementary Table 1) from where the curve was reproduced are shown. In the x-axis, the contents in the parentheses show the method to calculate CA (CD, number of chilling days in a specific temperature range; CH, number of chilling hours; CU11H, model C_{11} in this study but calculated at the hourly scale), temperature range (°C) and the starting date. In the y-axis, the contents in the parentheses show the method to calculate HR (GDD, growing degree days with a specific threshold temperature; GDH, growing degree hours; days to budburst if the original reference did not report the temperature), threshold temperature (°C) and the forcing daylength (constant daylength or outdoor photoperiod). BB: budburst date; Bloom: first flowering date. NRC, negative CA-HR relationship confirmed by the original references (but they did not report the curve). The blue color represents species with a negative relationship, while the red color represents species with a positive relationship.

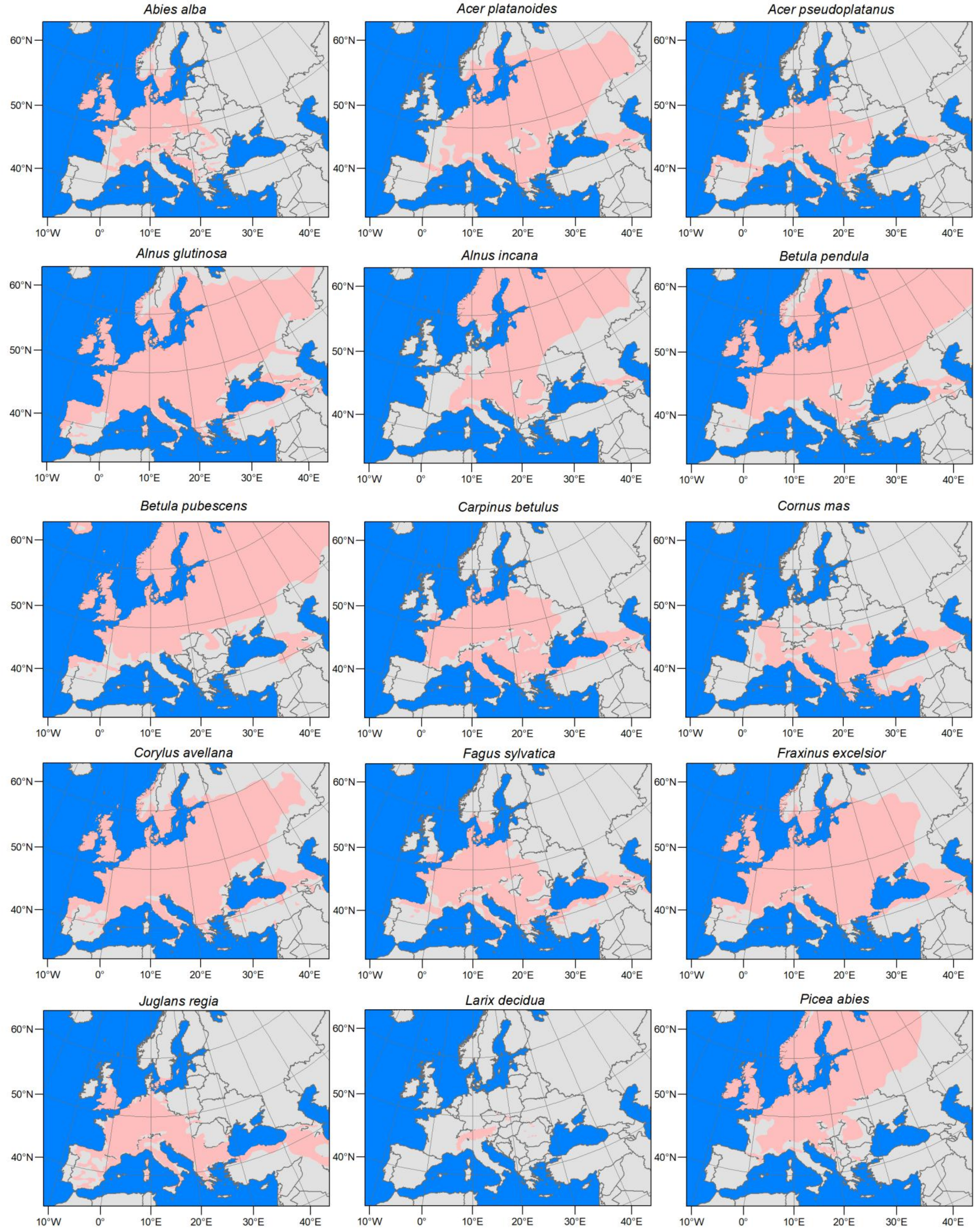

Supplementary Fig. 2 partial

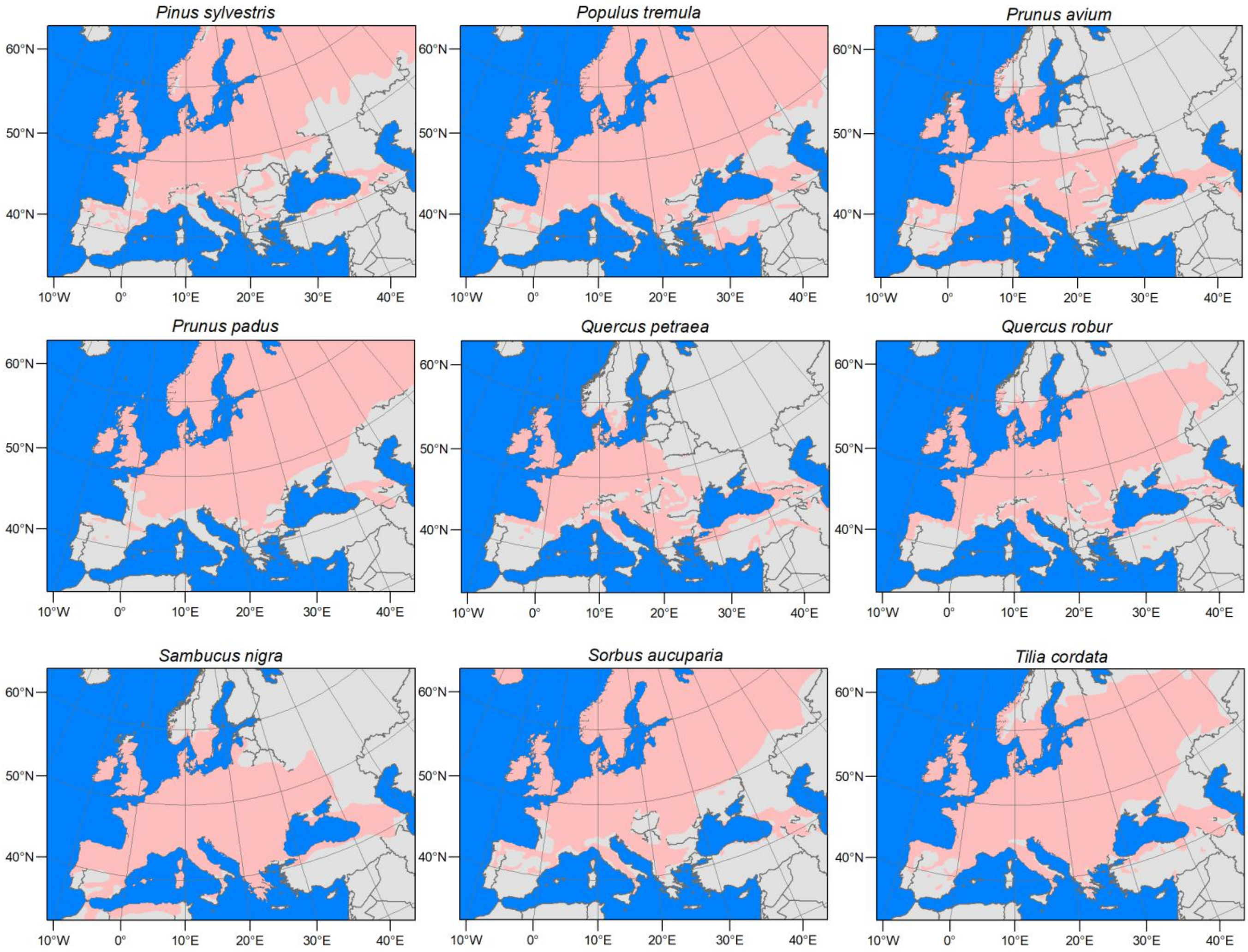

Supplementary Fig. 2 | Distribution of 24 main European woody species following the negative relationship between chilling accumulation and heat requirement. These species include the most dominant forest tree species in Europe, whose distribution area covers various habitats ranging from mountainous regions in southern and Eastern Europe to lowlands in central and northern Europe. The distribution data are from chorological maps for the main European woody species (https://doi.org/10.1016/j.dib.2017.05.007)

.

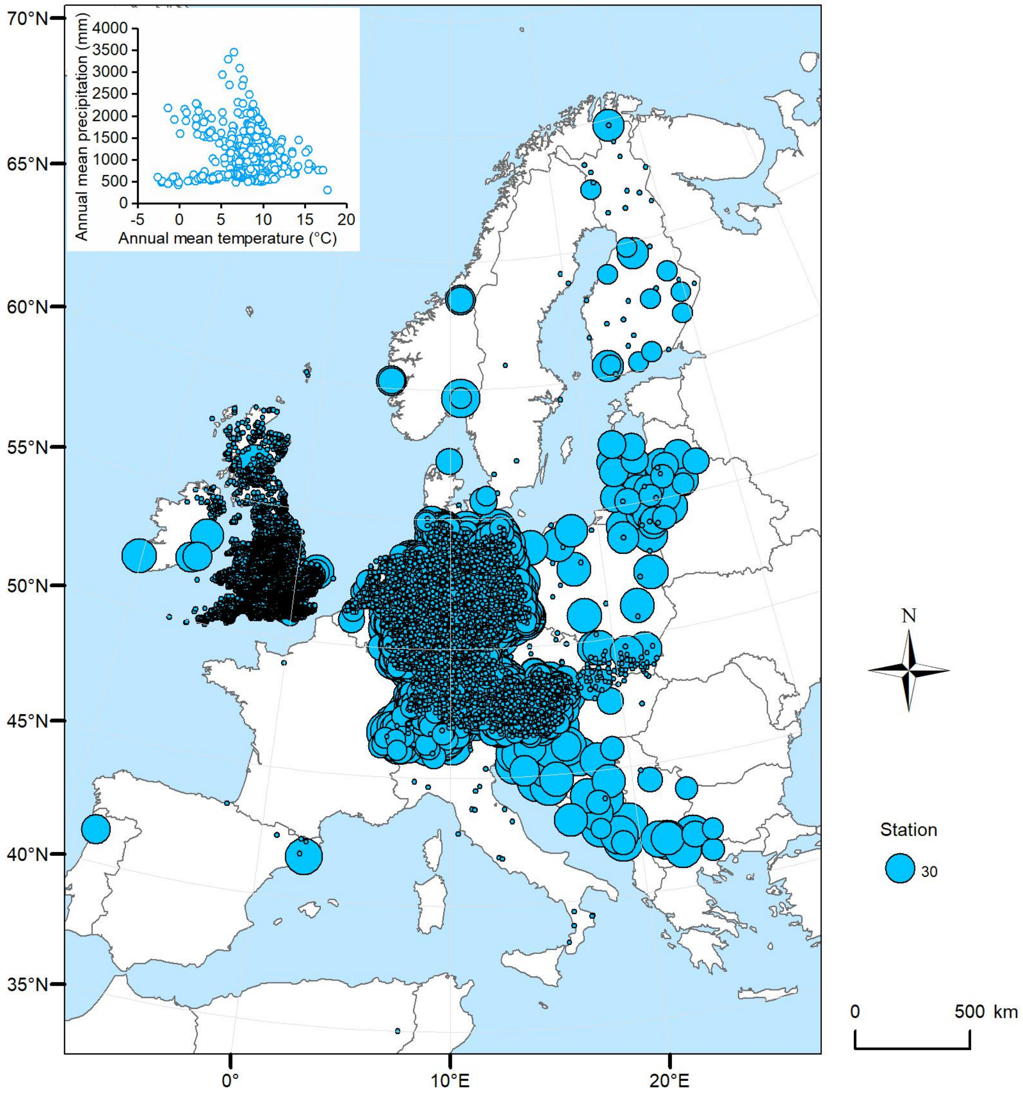

Supplementary Fig. 3 | Distribution of the phenological stations. Circle size: duration of phenological records (number of years) at each station. The left corner shows the annual mean temperature and precipitation of each phenological stations.

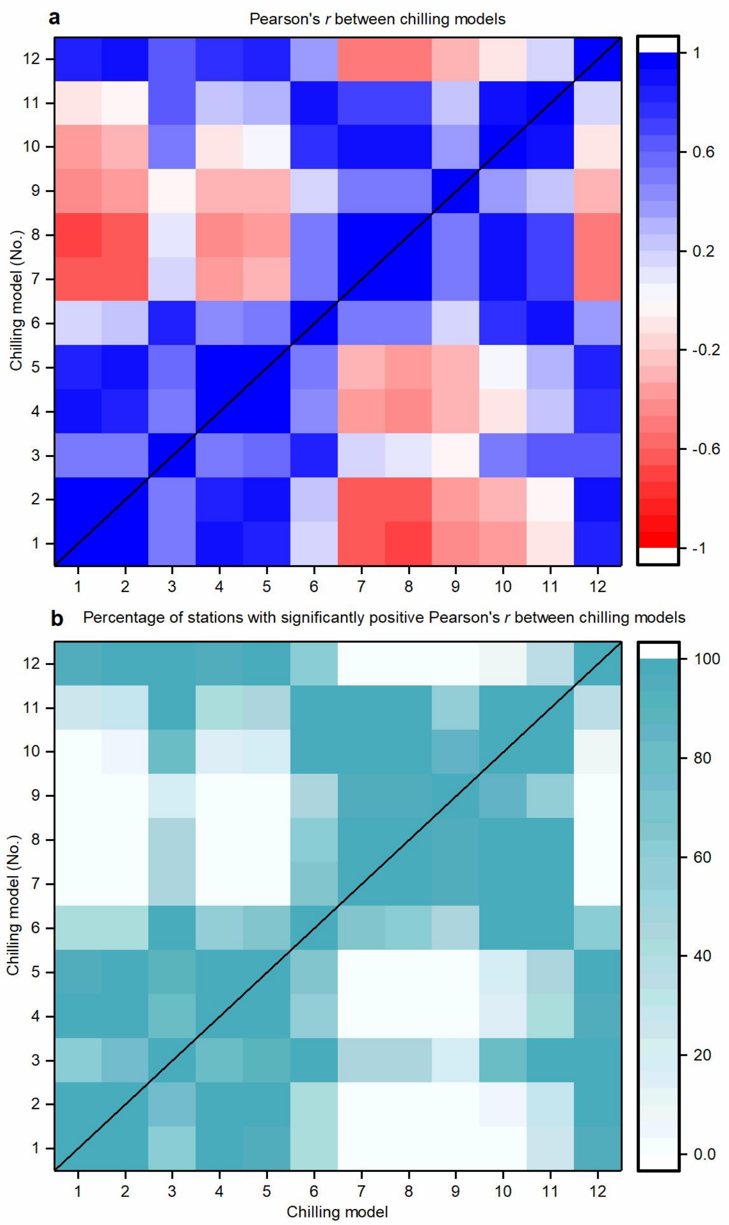

Supplementary Fig. 4 | Correlations between chilling accumulations calculated by using the different chilling models. **a**, Pearson's *r* among chilling accumulations calculated by each pair of chilling models averaged from all phenological stations in Supplementary Fig. 3**. b,** Percentage of stations with significantly positive Pearson's *r* (p<0.05) between chilling models.

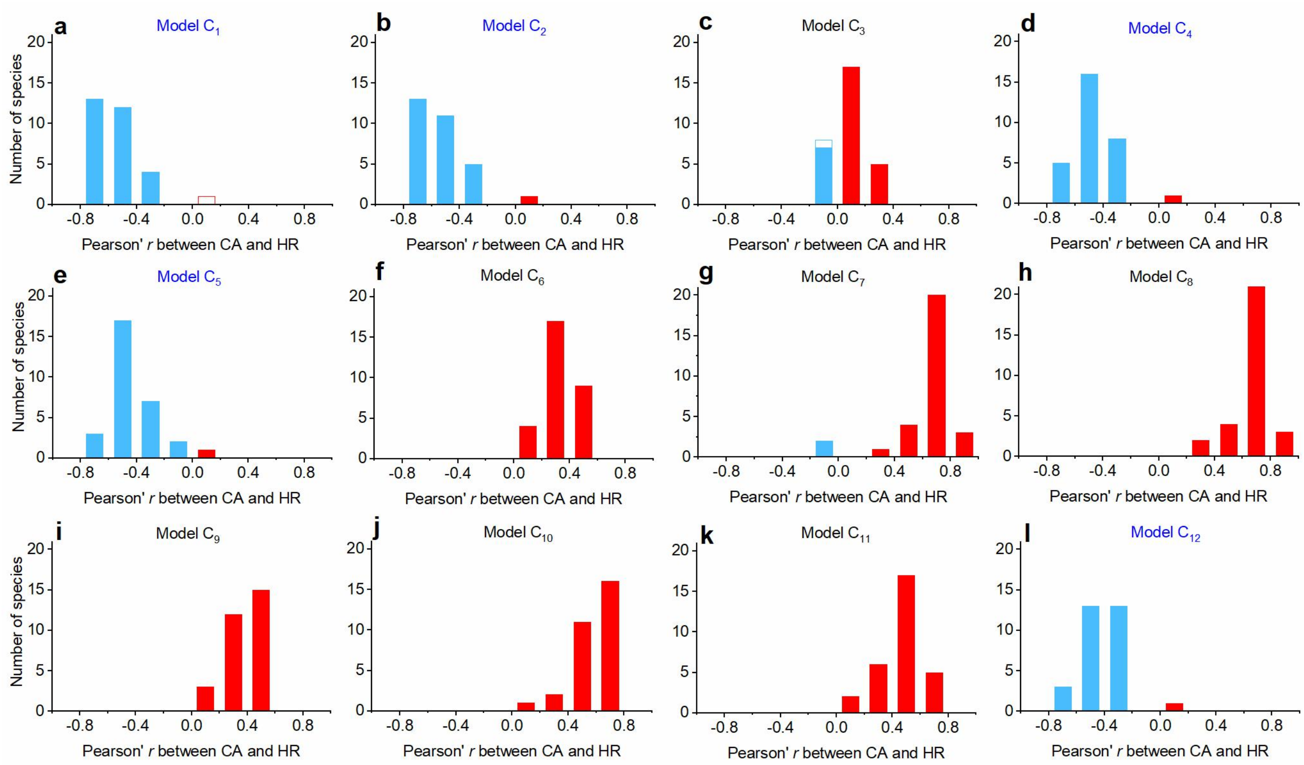

Supplementary Fig. 5 | Frequency distribution of Pearson's *r* between chilling accumulation and heat requirement. Chilling accumulation (CA) from 1 November to the onset of spring events was calculated for each species using the 12 chilling models (**a-l**), and the heat requirement (HR) was calculated as accumulated temperatures >0 °C from 1 January to the onset of spring events. Pearson's *r* between CA and HR was calculated using all records. The blue and red bars represent negative and positive Pearson's *r*, respectively. Solid bars indicate a significant Pearson's *r* at $p<0.05$ (two-sided *t*-test). The significantly negative Pearson's *r* between CA and HR were found in most species for Models C_1, C_2, C_4, C_5, and C_{12}, which supported the physiological assumption in Fig. 1.

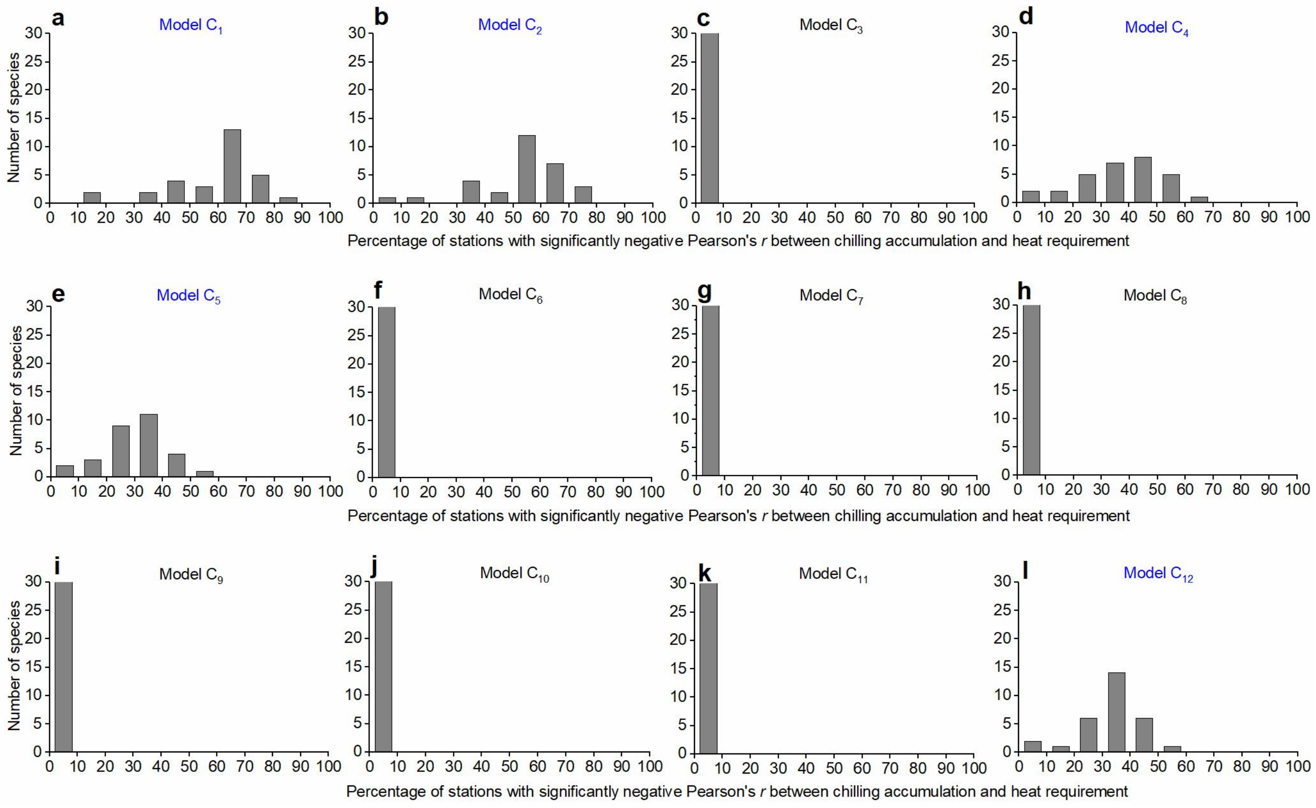

Supplementary Fig. 6 | Frequency distribution of the percentage of stations with significantly (p<0.05) negative Pearson's r between chilling accumulation and the heat requirement. Chilling accumulation (CA) from 1 November to the onset of spring event was calculated for each species using the 12 chilling models (**a-l**) s, and the heat requirement (HR) was calculated as accumulated temperatures >0 °C from 1 January to the onset of spring events. Pearson's r between CA and HR was calculated for each station that had at least 15 years of observations, and the percentages of significantly negative Pearson's r (p<0.05, two-sided t-test) were determined for each species. CA and HR were significantly and negatively correlated at >30% of the stations in most species for Models C_1, C_2, C_4, C_5, and C_{12}.

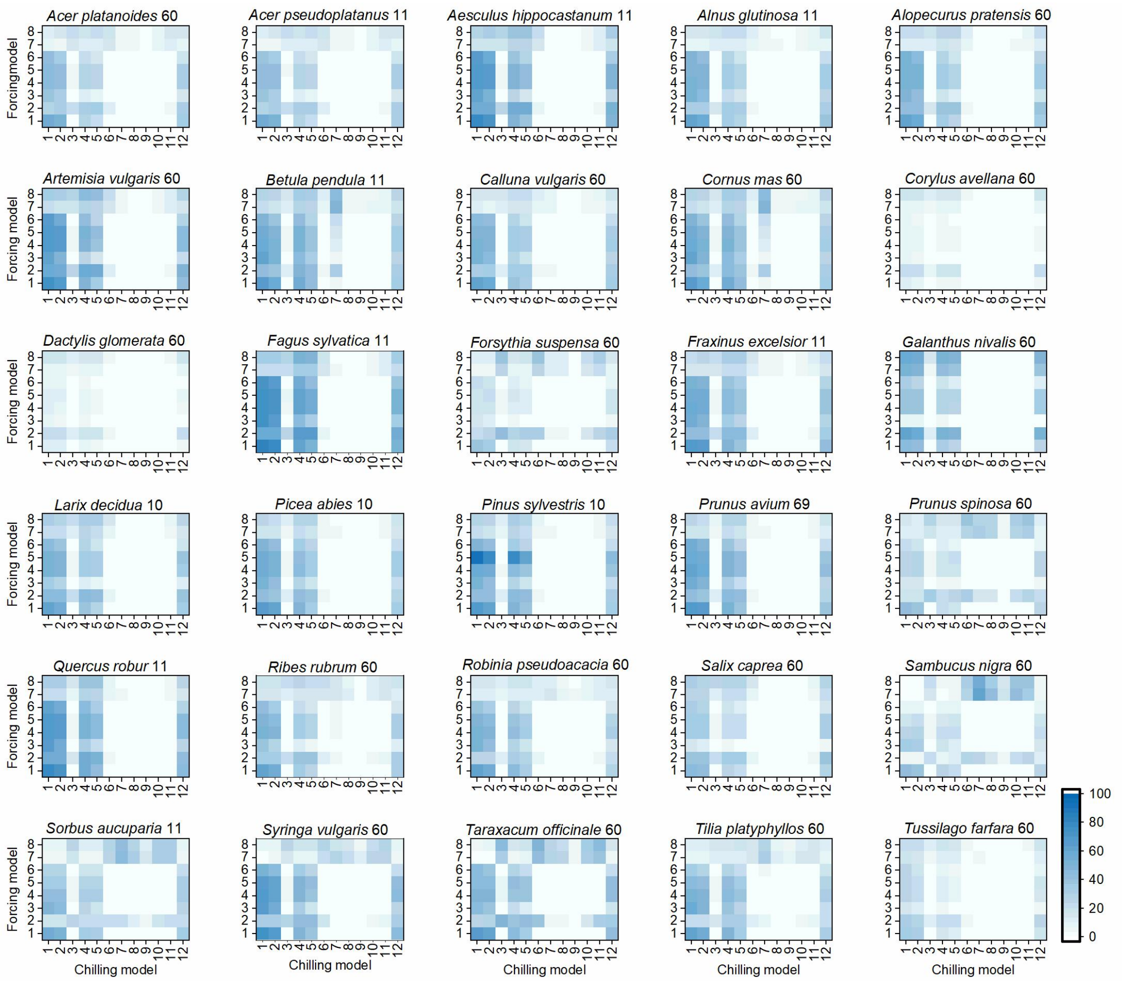

Supplementary Fig. 7 | Percentage of stations with significantly negative Pearson's *r* between chilling accumulation and the heat requirement. Chilling accumulation (CA) from 1 November to the onset of spring events was calculated for each species/event using the 12 chilling models, and the heat requirement (HR) from 1 January to the onset of spring events was calculated using the 8 forcing models. Pearson's *r* between CA and HR was calculated for each station that had at least 15 years of observations. The percentage of stations with significantly negative Pearson's *r* ($p<0.05$, two-sided *t*-test) between CA and HR is shown for each species/event. The events are coded using the BBCH scale: 10, first leaves separated; 11, first leaves unfolded; 60, first flowers open; and 69, end of flowering.

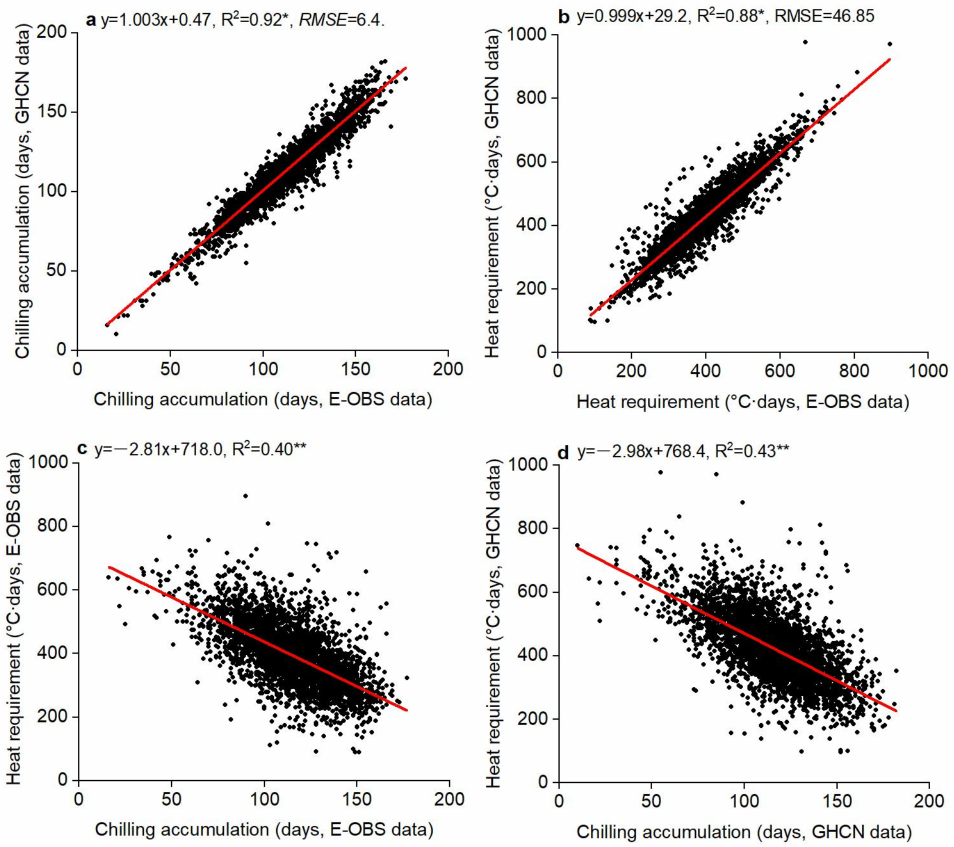

Supplementary Fig. 8 | Comparison between chilling accumulation (CA) and heat requirement (HR) for leaf-out of *Betula pendula* based on two temperature dataset. CA was calculated based on model C_1 accumulated from 1 November in the previous year to the leaf-out date. HR was calculated based on a commonly used forcing model (integrating daily mean temperatures >0 °C from 1 January to leaf-out date). The CA and HR in corresponding grid cells of E-OBS data and at corresponding stations of GHCN data are compared. **a**, CA between two datasets; **b**, HR between two datasets; **c**, CA-HR relationship for E-OBS data; **d**, CA-HR relationship for GHCN data. Root-mean-square error (RMSE) between the two datasets is shown. Also, the linear regression line (in red) and its R^2 are shown. A two-sided *F*-test is used to test whether the slope equals zero. n=3965. **: $p<0.01$.

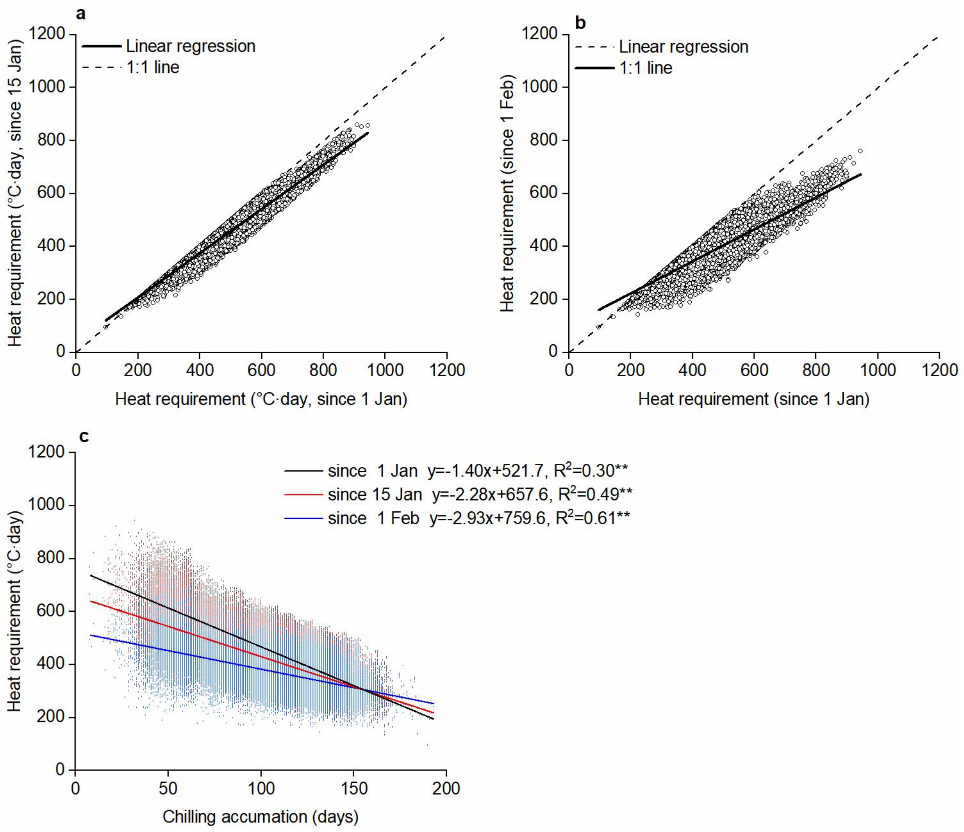

Supplementary Fig. 9 | Comparison between heat requirement (HR) for leaf-out of *Betula pendula* based on different starting dates of temperature accumulation. HR was calculated based on a commonly used forcing model (integrating daily mean temperatures >0 °C). **a**, HR accumulated from 1 January vs. from 15 January; **b**, HR accumulated from 1 January vs. from 1 February; **c**, chilling accumulation(CA)-HR relationship for different starting dates of temperature accumulation. CA was calculated based on model C_1 accumulated from 1 November in the previous year to the leaf-out date. The linear regression lines are shown (n=3965). A two-sided *F*-test is used to test whether the slope equals zero. **: $p<0.01$.

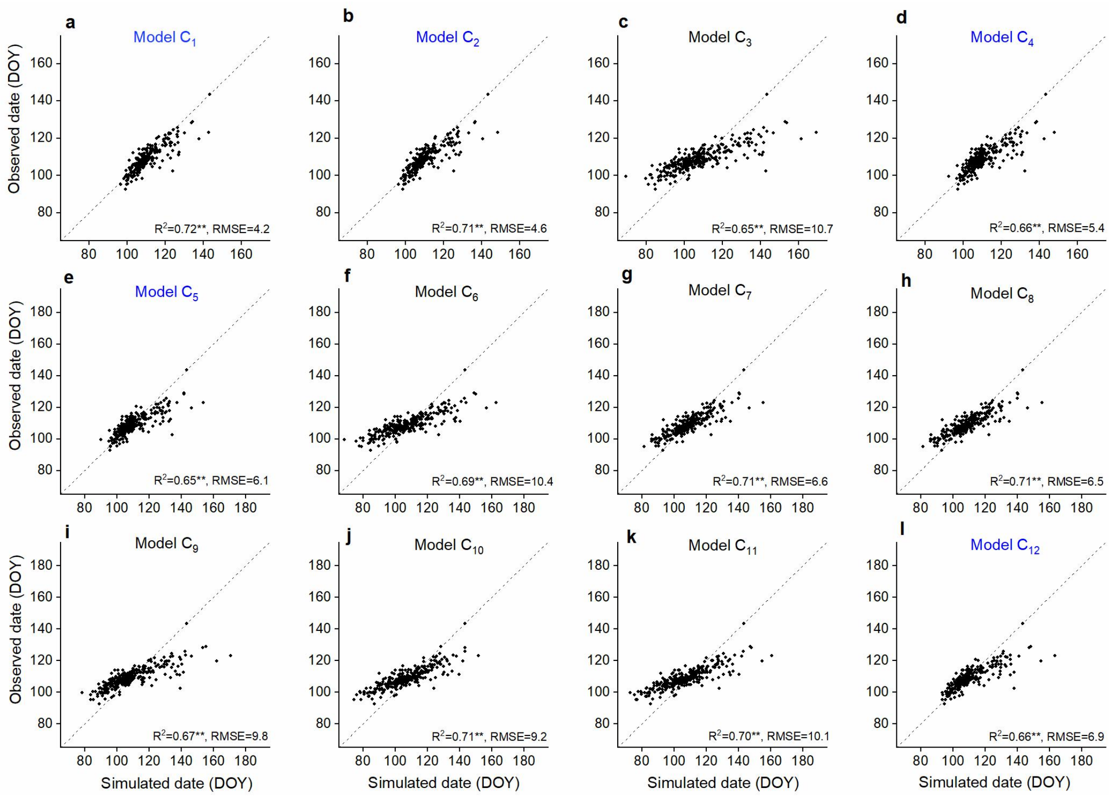

Supplementary Fig. 10 | Comparisons between observed and simulated leaf-out date of *Betula pendula* averaged from 1980 to 2018. Each point represents a 0.5° × 0.5° grid (n=303). Spring phenological events were simulated using the linear regression between chilling accumulations based on the 12 chilling models (from 1 November to the onset of spring events) and the heat requirement (the accumulated temperature >0 °C from 1 January to the onset of spring events) for all records (1951-2018). The past phenological change over 1980-2018 was simulated by using the E-OBS data. DOY, day of the year. The dashed line represents the 1:1 line. R^2: goodness of fit. A two-sided *F*-test is used to test whether the slope equals zero. **: $p<0.01$. RMSE: root-mean-square error (days).

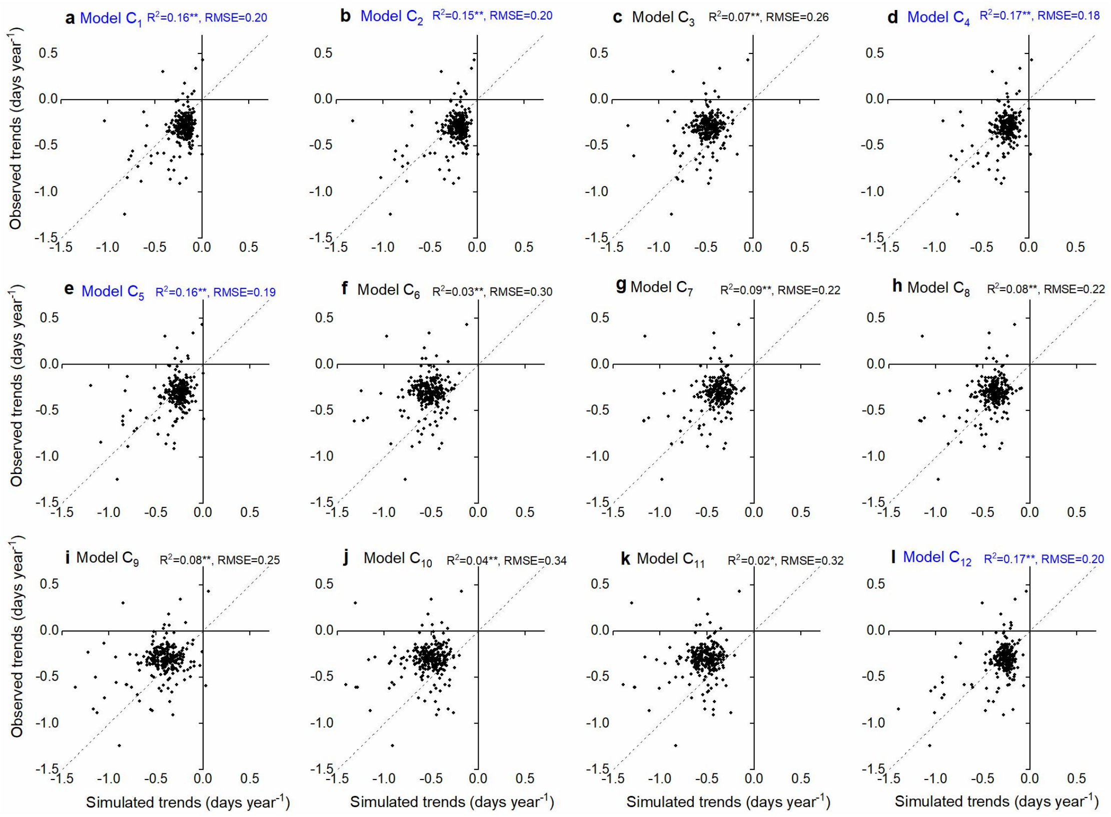

Supplementary Fig. 11 | Comparisons between observed and simulated trends in the leaf-out date of *Betula pendula* from 1980 to 2018. Trends was estimated as the slope of the linear regression of spring phenology against year for each 0.5° × 0.5° grid with at least 15-year observation data (n=265). Spring phenological events were simulated using the linear regression between chilling accumulations based on the 12 chilling models (from 1 November to the onset of spring events) and the heat requirement (the accumulated temperature >0 °C from 1 January to the onset of spring events) for all records (1951-2018). The past phenological change over 1980-2018 was simulated by using the E-OBS data. The dashed line represents the 1:1 line. R^2: goodness of fit. A two-sided F-test is used to test whether the slope equals zero. **: p<0.01. *: p<0.05. RMSE: root-mean-square error in days year^{-1}.

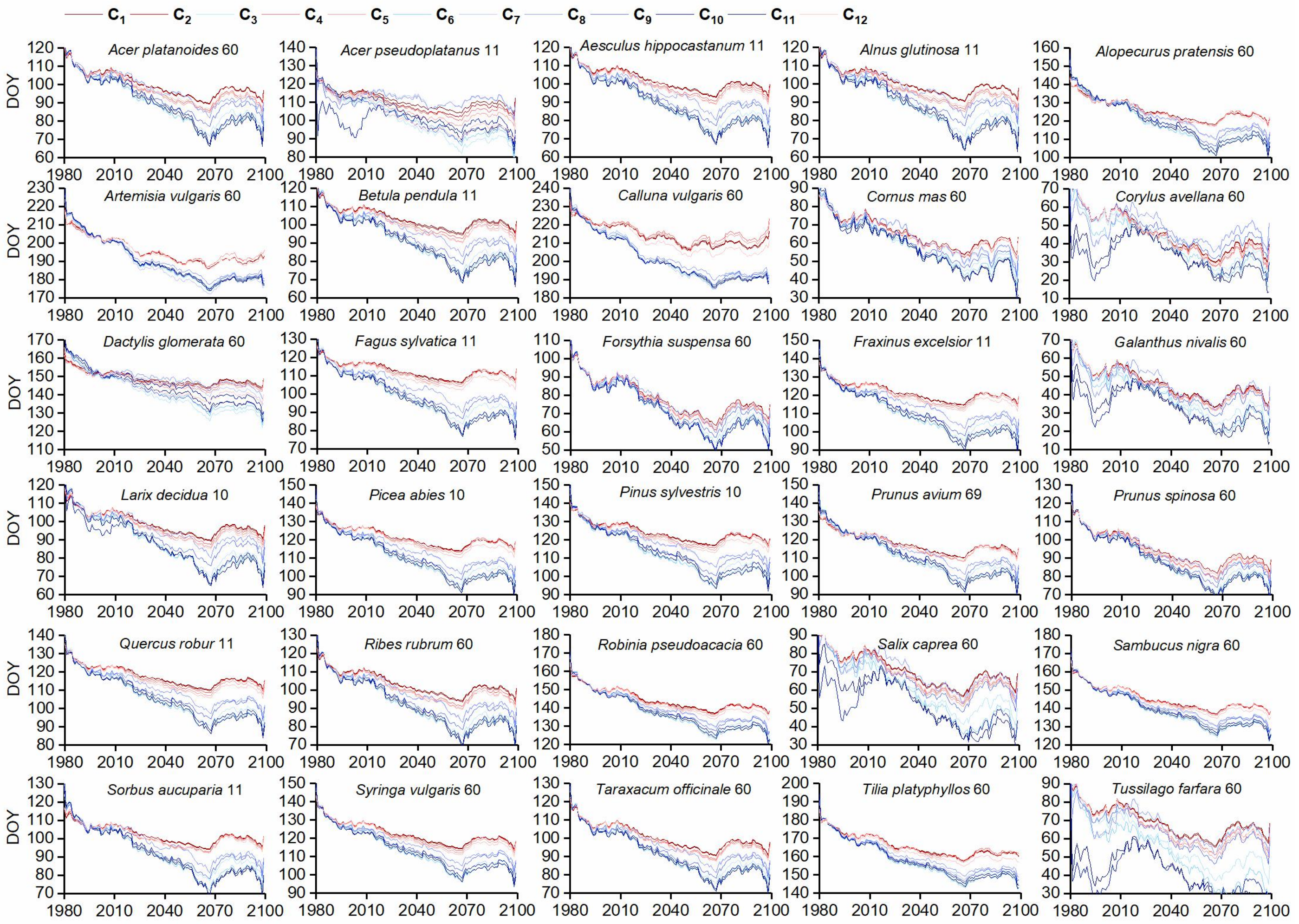

Supplementary Fig. 12 | Changes in spring phenology from 2019 to 2099 under the RCP 4.5 scenario. Spring phenological events were simulated using the linear regression between chilling accumulations based on the 12 chilling models (from 1 November to the onset of spring events) and the heat requirement (the accumulated temperature >0 °C from 1 January to the onset of spring events) for all records (1951-2018). The past phenological change over 1980-2018 was also simulated by using the E-OBS data. Each panel represents a spring event for one species. The events are coded using the BBCH scale: 10, first leaves separated; 11, first leaves unfolded; 60, first flowers open; and 69, end of flowering. DOY, day of the year. The dates of the spring phenological events were smoothed using an 11-year moving average. The red lines represent valid models (C_1, C_2, C_4, C_5, and C_{12}), and the blue lines represent invalid models.

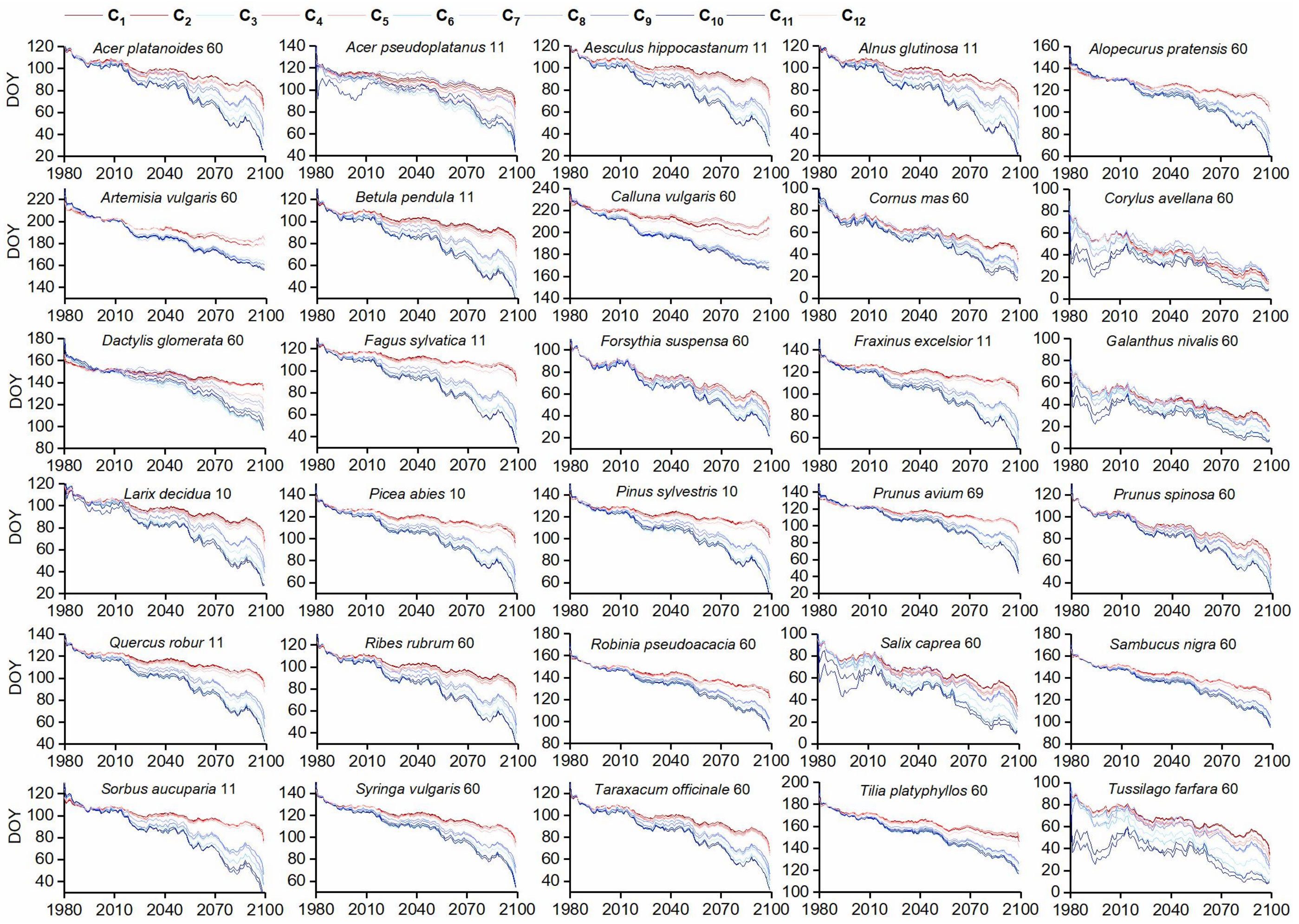

Supplementary Fig. 13 | Changes in spring phenology from 2012 to 2099 under the RCP 8.5 scenario. Spring phenological events were simulated using the linear regression between chilling accumulations based on the 12 chilling models (from 1 November to the onset of spring events) and the heat requirement (the accumulated temperature >0 °C from 1 January to the onset of spring events) for all records (1951-2018). The past phenological change over 1980-2018 was also simulated by using the E-OBS data. Each panel represents a spring event for one species. The events are coded using the BBCH scale: 10, first leaves separated; 11, first leaves unfolded; 60, first flowers open; and 69, end of flowering. DOY, day of the year. The dates of the spring phenological events were smoothed using an 11-year moving average. The red lines represent valid models (C_1, C_2, C_4, C_5, and C_{12}), and the blue lines represent invalid models.

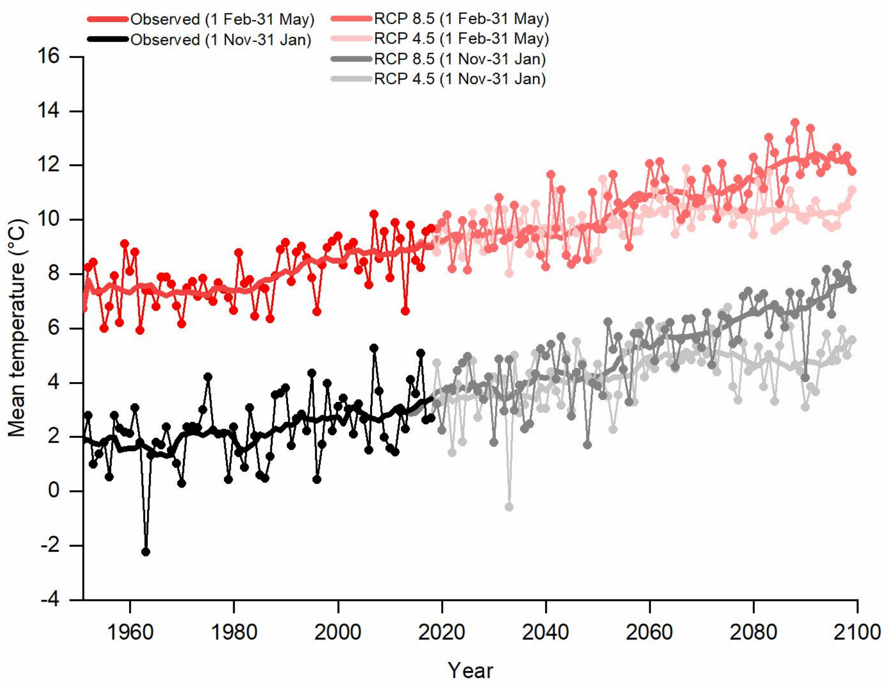

Supplementary Fig. 14 | Temperature change in Europe from 1951 to 2099. For the past period (1951-2018), temperature data is from the E-OBS data. For future climatic data (2019-2099), we used the data simulated by the HADGEM2-ES model. The temperature during the chilling period (black line) is averaged from the previous 1 November to 31 January, while the temperature during the forcing period (red line) is averaged from 1 February to 31 May. The bold lines show the result of the 11-year moving average.

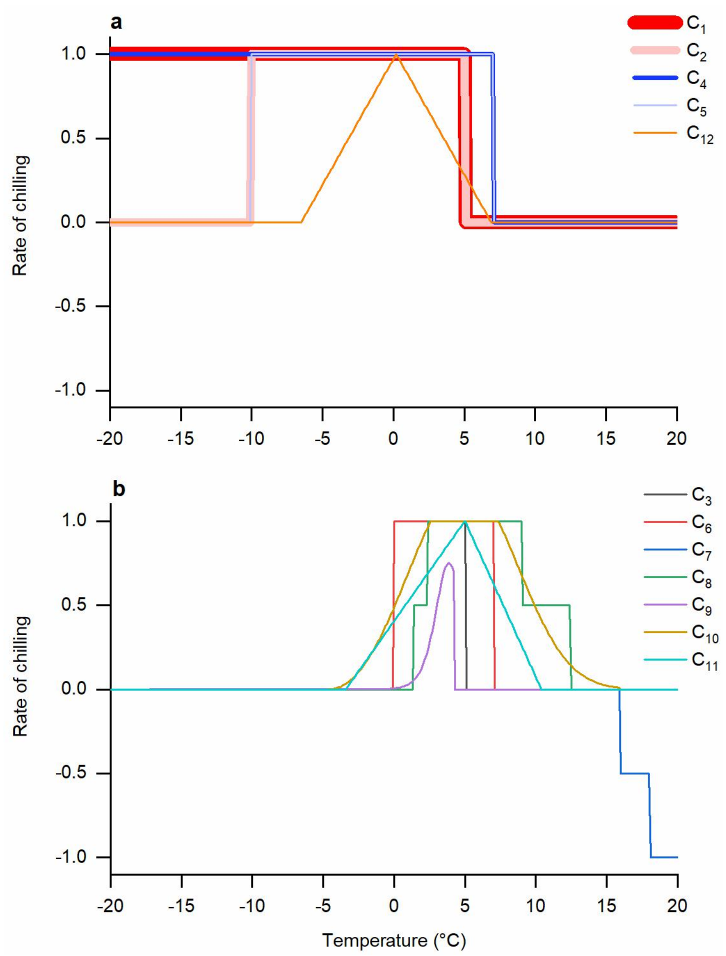

Supplementary Fig. 15 | Response of the rate of chilling for the 12 chilling models. **a,** Valid models. **b,** Invalid models.

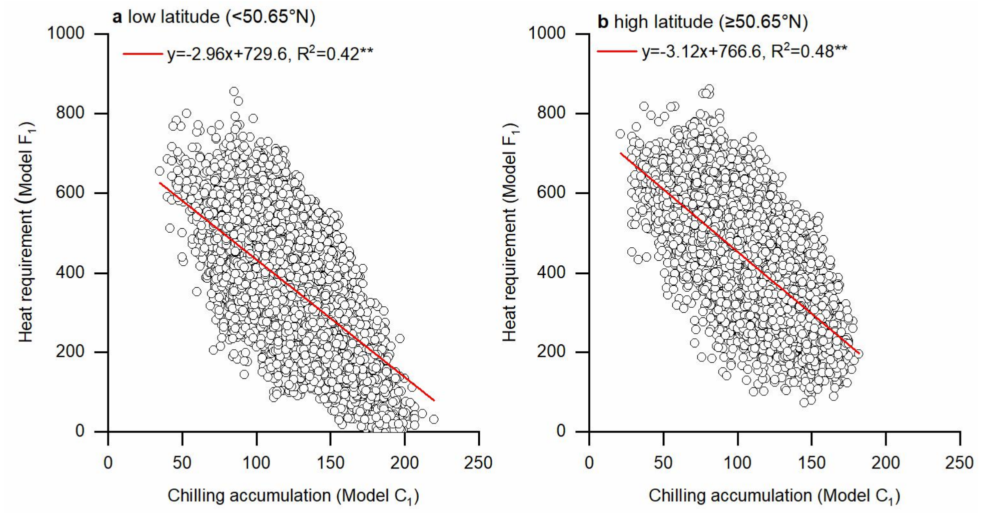

Fig. 16 Relationship between chilling accumulation (CA) and heat requirement (HR) for leaf-out of *Betula pendula* at different latitudes. **a**, at latitudes lower than 50.65 °N. **b**, at latitudes higher than 50.65 °C. The red line represents the linear regression line (n=32275). A two-sided *F*-test is used to test whether the slope equals zero. **: p<0.01.

**Supplementary Table 1 | ** Examples of studies examining the correlation between chilling accumulation and heat requirement of spring phenology.

Year	Species	Event	Region	Method	Ref
1983	*Picea sitchensis*	Budburst	UK	Observation	[1]
1989	15 species	Budburst	UK	Experiment	[2]
1993	9 species	Budburst	Norway	Experiment	[3]
1993	*Carya illinoinensis*	Budburst	USA	Experiment	[4]
1995	2 species	Bud-burst	Norway	Experiment	[5]
2005	2 species	Leaf unfolding	Norway	Experiment	[6]
2009	*Euphorbia elula*	Flowering	USA	Experiment	[7]
2010	*Euphorbia elula*	Flowering	USA	Experiment	[8]
2010	*Pseudotsuga menziesii*	Budburst	USA	Experiment	[9]
2011	4 species	Budburst	UK	Experiment	[10]
2011	*Prunus armeniaca*	Budburst	South Africa	Experiment	[11]
2011	*Prunus persica*	Budburst	USA	Experiment	[12]
2011	*Betula pubescens*	Budburst	UK	Experiment	[13]
2013	3 species	Leaf unfolding	Belgium	Experiment	[14]
2013	2 species	Leaf unfolding	France	Observation	[15]
2014	36 species	Budburst	Germany	Experiment	[16]
2014	*Picea abies*	Budburst	Finland	Experiment	[17]
2015	13 species	Leaf unfolding	Europe	Observation	[18]
2015	2 species	Budburst	UK	Experiment	[19]
2017	7 species	Budburst	Canada	Experiment	[20]
2017	6 species	Budburst	USA	Experiment	[21]
2018	28 species	Budburst	USA, Canada	Experiment	[22]
2019	2 species	Leaf unfolding	Belgium	Experiment	[23]
2019	*Leymus chinensis*	Leaf unfolding	China	Experiment	[24]

Supplementary Table 2 | List of 91 perennials with negative correlations between chilling accumulation and the heat requirement of spring phenology.

Plant type	Number	Species
Deciduous broadleaved shrub	23	*Acer tataricum*, *Aronia melanocarpa*, *Cornus alba*, *Cornus mas*, *Corylus avellana*, *Crataegus monogyna*, *Hamamelis virginiana*, *Ilex mucronatus*, *Kalmia angustifolia*, *Lonicera canadensis*, *Lyonia ligustrina*, *Rhamnus frangula*, *Rhododendron prinophyllum*, *Rosa rugosa*, *Rubus idaeus*, *Salix viminalis*, *Spiraea alba*, *Symphoricarpos albus*, *Syringa chinensis*, *Syringa vulgaris*, *Vaccinium myrtilloides*, *Viburnum cassinoides*, *Viburnum lantanoides*
Deciduous broadleaved tree	52	*Acer negundo, Acer pensylvanicum, Acer platanoides, Acer pseudoplatanus, Acer rubrum, Acer saccharum, Aesculus hippocastanum, Alnus glutinosa, Alnus incana, Alnus incana subsp. rugosa, Betula alleghaniensis, Betula lenta, Betula papyrifera, Betula pendula, Betula pubescens, Carpinus betulus, Carya illinoinensis, Fagus grandifolia, Fagus sylvatica, Fraxinus chinensis, Fraxinus excelsior, Fraxinus nigra, Juglans ailantifolia, Juglans cinerea, Juglans regia, Malus sp.(apple), Nyssa sylvatica, Populus balsamifera, Populus deltoides, Populus grandidentata, Populus tremula, Populus tremuloides, Populus trichocarpa, Prunus armeniaca (apricot), Prunus avium, Prunus padus, Prunus pensylvanica, Prunus persica (Peach), Prunus serotina, Pyrus communis(pear), Quercus alba, Quercus bicolor, Quercus ellipsoidalis, Quercus petraea, Quercus robur, Quercus rubra, Quercus velutina, Robinia pseudoacacia, Salix × smithiana, Sambucus nigra, Sorbus aucuparia, Tilia cordata*
Deciduous coniferous tree	2	*Larix decidua, Larix laricina*
Evergreen coniferous tree	12	*Abies alba*, *Tsuga heterophylla*, *Picea abies*, *Picea glauca*, *Picea mariana*, *Picea sitchensis*, *Pinus banksiana*, *Pinus contorta*, *Pinus strobus*, *Pinus sylvestris*, *Pinus wallichiana*, *Pseudotsuga menziesil*
Herbaceous perennial	2	*Leymus chinensis*, *Euphorbia elula*

All perennials are extracted from the studies listed in Supplementary Table 1.

Supplementary Table 3 | Summary of species and spring events investigated in this study.

No.	Species	BBCH	Plant type	No. of stations	No. of records
1	*Acer platanoides**	60	Tree	2674	80084
2	*Acer pseudoplatanus**	11	Tree	76	1578
3	*Aesculus hippocastanum**	11	Tree	3942	129530
4	*Alnus glutinosa**	11	Tree	2477	72152
5	*Alopecurus pratensis*	60	Herb	1059	23072
6	*Artemisia vulgaris*	60	Herb	800	15953
7	*Betula pendula**	11	Tree	3912	127990
8	*Calluna vulgaris*	60	Shrub	2655	80681
9	*Cornus mas**	60	Shrub	40	1271
10	*Corylus avellana**	60	Shrub	3466	108820
11	*Dactylis glomerata*	60	Herb	225	5243
12	*Fagus sylvatica**	11	Tree	3321	103450
13	*Forsythia suspense*	60	Shrub	3023	92325
14	*Fraxinus excelsior**	11	Tree	2415	72315
15	*Galanthus nivalis*	60	Herb	4178	139530
16	*Larix decidua**	10	Tree	2968	92415
17	*Picea abies**	10	Tree	3185	100960
18	*Pinus sylvestris**	10	Tree	2699	78768
19	*Prunus avium**	69	Tree	684	12887
20	*Prunus spinosa*	60	Shrub	3013	97249
21	*Quercus robur**	11	Tree	3394	106650
22	*Ribes rubrum*	60	Shrub	3359	100030
23	*Robinia pseudoacacia**	60	Tree	2638	77764
24	*Salix caprea*	60	Shrub	3828	125450
25	*Sambucus nigra**	60	Shrub	4115	129710
26	*Sorbus aucuparia**	11	Shrub	1192	25877
27	*Syringa vulgaris**	60	Shrub	4177	139020
28	*Taraxacum officinale*	60	Herb	3954	130740
29	*Tilia platyphyllos*	60	Tree	3351	105330
30	*Tussilago farfara*	60	Herb	3742	116800

The BBCH scale is used to identify the phenological events of plants: 10, first leaves separated; 11, first leaves unfolded; 60, first flowers open; and 69, end of flowering. *: the species which have been proved to have a negative relationship between HR and CA based on previous studies.

References

1. Cannell, M. G. R. & Smith, R. I., Thermal time, chill days and prediction of budburst in *Picea sitchensis*. *J. Appl. Ecol.* **20**, 951-963 (1983).
2. Murray, M. B., Cannell, M. & Smith, R. I., Date of budburst of fifteen tree species in Britain following climatic warming. *J. Appl. Ecol.* **26**, 693-700 (1989).
3. Heide, O. M., Daylength and thermal time responses of budburst during dormancy release in some northern deciduous trees. *Physiol. Plantarum* **88**, 531-540 (1993).
4. Sparks, D., Chilling and heating model for pecan budbreak. *J. Am. Soc. Hortic. Sci.* **118**, 29-35 (1993).
5. Myking, T. & Heide, O. M., Dormancy release and chilling requirement of buds of latitudinal ecotypes of *Betula pendula* and *B. pubescens*. *Tree Physiol.* **15**, 697-704 (1995).
6. Heide, O. M. & Prestrud, A. K., Low temperature, but not photoperiod, controls growth cessation and dormancy induction and release in apple and pear. *Tree Physiol.* **25**, 109-114 (2005).
7. Foley, M. E., Anderson, J. V. & Horvath, D. P., The effects of temperature, photoperiod, and vernalization on regrowth and flowering competence in *Euphorbia esula* (Euphorbiaceae) crown buds. *Botany* **87**, 986-992 (2009).
8. Dogramaci, M. et al., Low temperatures impact dormancy status, flowering competence, and transcript profiles in crown buds of leafy spurge. *Plant Mol. Biol.* **73**, 207-226 (2010).
9. Harrington, C. A., Gould, P. J. & St Clair, J. B., Modeling the effects of winter environment on dormancy release of Douglas-fir. *Forest Ecol. Manag.* **259**, 798-808 (2010).
10. Caffarra, A. & Donnelly, A., The ecological significance of phenology in four different tree species: effects of light and temperature on bud burst. *Int. J. Biometeorol.* **55**, 711-721 (2011).
11. Campoy, J. A., Ruiz, D., Cook, N., Allderman, L. & Egea, J., High temperatures and time to budbreak in low chill apricot 'Palsteyn'. Towards a better understanding of chill and heat requirements fulfilment. *Sci. Hortic.-Amsterdam* **129**, 649-655 (2011).
12. Okie, W. R. & Blackburn, B., Increasing chilling reduces heat requirement for floral budbreak in Peach. *Hortscience* **46**, 245-252 (2011).
13. Caffarra, A., Donnelly, A., Chuine, I. & Jones, M. B., Modelling the timing of Betula pubescens budburst. I. Temperature and photoperiod: a conceptual model. *Clim. Res.* **46**, 147-157 (2011).
14. Fu, Y. H., Campioli, M., Deckmyn, G. & Janssens, I. A., Sensitivity of leaf unfolding to experimental warming in three temperate tree species. *Agr. Forest Meteorol.* **181**, 125-132 (2013).
15. Vitasse, Y. & Basler, D., What role for photoperiod in the bud burst phenology of European beech. *Eur. J. Forest Res.* **132**, 1-8 (2013).
16. Laube, J. et al., Chilling outweighs photoperiod in preventing precocious spring development. *Global Change Biol.* **20**, 170-182 (2014).
17. Vihera-Aarnio, A., Sutinen, S., Partanen, J. & Hakkinen, R., Internal development of vegetative buds of Norway spruce trees in relation to accumulated chilling and forcing temperatures. *Tree Physiol.* **34**, 547-556 (2014).
18. Fu, Y. H. et al., Increased heat requirement for leaf flushing in temperate woody species over 1980-2012: effects of chilling, precipitation and insolation. *Global Change Biol.* **21**, 2687-2697 (2015).
19. Pletsers, A., Caffarra, A., Kelleher, C. T. & Donnelly, A., Chilling temperature and photoperiod influence the timing of bud burst in juvenile *Betula pubescens* Ehrh. and *Populus tremula* L. trees.

Ann. Forest Sci. **72**, 941-953 (2015).

20. Man, R., Lu, P. & Dang, Q., Insufficient chilling effects vary among boreal tree species and chilling duration. *Front. Plant Sci.* **8**, 1354 (2017).
21. Nanninga, C., Buyarski, C. R., Pretorius, A. M. & Montgomery, R. A., Increased exposure to chilling advances the time to budburst in North American tree species. *Tree Physiol.* **37**, 1727-1738 (2017).
22. Flynn, D. F. B. & Wolkovich, E. M., Temperature and photoperiod drive spring phenology across all species in a temperate forest community. *New Phytol.* **219**, 1353-1362 (2018).
23. Fu, Y. H. et al., Short photoperiod reduces the temperature sensitivity of leaf-out in saplings of *Fagus sylvatica* but not in horse chestnut. *Global Change Biol.* **25**, 1696-1703 (2019).
24. Huang, W., Ge, Q., Wang, H. & Dai, J., Effects of multiple climate change factors on the spring phenology of herbaceous plants in Inner Mongolia, China: Evidence from ground observation and controlled experiments. *Int. J. Climatol.* **39**, 5140-5153 (2019).
