# Supplementary material for: Overestimation of the effect of climatic warming on spring phenology due to misrepresentation of chilling
Source: Nat Commun. 2020 Oct 2;11:4945. doi: 10.1038/s41467-020-18743-8 (PMC7532433; doi:10.1038/s41467-020-18743-8)
Supplement: Supplementary file 3 — Reporting Summary [file 41467_2020_18743_MOESM3_ESM.pdf]

nature research

Corresponding author(s): Huanjiong Wang

Last updated by author(s): Aug 12, 2020

Reporting Summary

Nature Research wishes to improve the reproducibility of the work that we publish. This form provides structure for consistency and transparency in reporting. For further information on Nature Research policies, see Authors & Referees and the Editorial Policy Checklist.

Statistics

For all statistical analyses, confirm that the following items are present in the figure legend, table legend, main text, or Methods section.

n/a	Confirmed	
☐	☒	The exact sample size (*n*) for each experimental group/condition, given as a discrete number and unit of measurement
☐	☒	A statement on whether measurements were taken from distinct samples or whether the same sample was measured repeatedly
☐	☒	The statistical test(s) used AND whether they are one- or two-sided<br>*Only common tests should be described solely by name; describe more complex techniques in the Methods section.*
☒	☐	A description of all covariates tested
☒	☐	A description of any assumptions or corrections, such as tests of normality and adjustment for multiple comparisons
☐	☒	A full description of the statistical parameters including central tendency (e.g. means) or other basic estimates (e.g. regression coefficient) AND variation (e.g. standard deviation) or associated estimates of uncertainty (e.g. confidence intervals)
☐	☒	For null hypothesis testing, the test statistic (e.g. *F*, *t*, *r*) with confidence intervals, effect sizes, degrees of freedom and *P* value noted<br>*Give P values as exact values whenever suitable.*
☒	☐	For Bayesian analysis, information on the choice of priors and Markov chain Monte Carlo settings
☐	☒	For hierarchical and complex designs, identification of the appropriate level for tests and full reporting of outcomes
☐	☒	Estimates of effect sizes (e.g. Cohen's *d*, Pearson's *r*), indicating how they were calculated

Our web collection on statistics for biologists contains articles on many of the points above.

Software and code

Policy information about availability of computer code

Data collection	No software was used for data collection.
Data analysis	We used MATLAB version 2015b to run all the analyses. All Matlab scripts, from initial processing of data sets to final analyses, are archived online at https://ww2.mathworks.cn/matlabcentral/fileexchange/74606-code-for-chilling-and-forcing-model.

For manuscripts utilizing custom algorithms or software that are central to the research but not yet described in published literature, software must be made available to editors/reviewers. We strongly encourage code deposition in a community repository (e.g. GitHub). See the Nature Research guidelines for submitting code & software for further information.

Data

Policy information about availability of data

All manuscripts must include a data availability statement. This statement should provide the following information, where applicable:

- Accession codes, unique identifiers, or web links for publicly available datasets
- A list of figures that have associated raw data
- A description of any restrictions on data availability

Phenological data can be accessed from http://www.pep725.eu/. E-OBS data set can be accessed from https://www.ecad.eu/download/ensembles/download.php. GHCH data set can be accessed from https://www.ncdc.noaa.gov/data-access/land-based-station-data/land-based-datasets/global-historical-climatology-network-ghcn. Future climate data (2019-2099) were available on https://esg.pik-potsdam.de/projects/isimip2b/.

Field-specific reporting

Please select the one below that is the best fit for your research. If you are not sure, read the appropriate sections before making your selection.

☐ Life sciences ☐ Behavioural & social sciences ☒ Ecological, evolutionary & environmental sciences

For a reference copy of the document with all sections, see nature.com/documents/nr-reporting-summary-flat.pdf

Ecological, evolutionary & environmental sciences study design

All studies must disclose on these points even when the disclosure is negative.

Study description	Spring warming substantially advanced leaf unfolding and flowering time for perennials. Winter warming, however, decreased chilling accumulation (CA), which increased the heat requirement (HR) and acted to delay spring phenology. Using long-term observations of leaf unfolding and flowering for 30 perennials at 15533 stations in Europe, we examined whether or not this CA-HR mechanism has been correctly interpreted in ecosystem models. We show that more than half (7 of 12) of current chilling models wrongly interpret the negative relationship between CA and HR, possibly because these invalid models overlook the effect of freezing temperature on dormancy release. Overestimation of the advance in spring phenology by the end of this century by these invalid chilling models could be as large as 7.6 and 20.0 days under RCPs 4.5 and 8.5, respectively. Our results highlight the need for a better representation of chilling for the correct understanding of spring phenological responses to future climate change.
Research sample	We used data from the Pan European Phenology Project PEP725, an open-access database with long-term plant phenological observations across 25 European countries (http://www.pep725.eu/). We selected 30 species for which sufficient phenological data were available, including 21 deciduous broadleaved trees or shrubs, 6 herbaceous perennials, 2 evergreen coniferous trees, and 1 deciduous coniferous tree (Table S3)
Sampling strategy	Because the analyzed data in our study were obtained from open-access database instead of designed experiments, sampling is not applicable to our study.
Data collection	The phenological data was from the Pan European Phenology Project. The data provided by this project is recorded by volunteers from 32 European meteorological services and project partners. They collected the phenological data following a standardized guidelines (BBCH code).
Timing and spatial scale	We used phenological data (mainly distributed in Central Europe, see Figure S1) from 1951 to 2018 with a precise location (latitude and longitude) for each plant. We used the E-OBS v19.0eHOM data set (daily mean, maximum, and minimum temperature, 1950-2018) with a spatial resolution of 0.1×0.1° for matching the phenological data. For future climatic data (2019-2099), we used daily minimum and maximum temperatures simulated by the HADGEM2-ES model (with a spatial resolution of 0.5×0.5°) under two climatic scenarios (RCP 4.5 and RCP 8.5).
Data exclusions	No data were excluded from the data sets in all the analyses.
Reproducibility	This is a study based on observation data, so we did not verify the reproducibility of experimental findings.
Randomization	Because the analyzed data in our study were obtained from open-access database instead of designed experiments, randomization is not applicable to our study.
Blinding	This is a study based on observation data, so blinding is not relevant to this study.

Did the study involve field work? ☐ Yes ☒ No

Reporting for specific materials, systems and methods

We require information from authors about some types of materials, experimental systems and methods used in many studies. Here, indicate whether each material, system or method listed is relevant to your study. If you are not sure if a list item applies to your research, read the appropriate section before selecting a response.

Materials & experimental systems

n/a	Involved in the study
☒	☐ Antibodies
☒	☐ Eukaryotic cell lines
☒	☐ Palaeontology
☒	☐ Animals and other organisms
☒	☐ Human research participants
☒	☐ Clinical data

Methods

n/a	Involved in the study
☒	☐ ChIP-seq
☒	☐ Flow cytometry
☒	☐ MRI-based neuroimaging
